# Association of hormone replacement therapy and image-detected breast cancer

**DOI:** 10.3389/fonc.2025.1647152

**Published:** 2025-10-01

**Authors:** Harry B. Burke

**Affiliations:** Department of Medicine, Uniformed Services University of the Health Sciences, Bethesda, MD, United States

**Keywords:** breast cancer, mammography, hormone replacement therapy, endocrine therapy, early detection, cancer screening

There has been a great deal of interest in the relationship between hormone replacement therapy (HRT) and image-detected breast cancer and it has been suggested that HRT causes breast cancer (1). In this paper, I summarize the key information regarding the relationship between HRT and breast cancer and I present an alternative view of HRT’s role in breast cancer. I suggest that HRT enhances the image of estrogen-receptor (ER) positive breast cancer which makes it easier to detect by mammographic screening, resulting in more early detected breast cancers because of lead time bias.

First, HRT does not cause breast cancer in all women. Most women taking HRT are not diagnosed with breast cancer. In the Million Woman Study (2), the women on HRT had 19 additional image-detected breast cancer cases per 1000 women over 10 years. Furthermore, after stopping HRT, the rate of image-detected breast cancer decreases and returns to baseline after approximately five years (3).

Second, women taking HRT have more abnormal mammograms because of their increased breast density due to the HRT (4) and they receive an increased evaluation of the presence of incident breast cancer (5) – resulting in the earlier detection of disease. HRT users are younger and have a lower stage of disease than the non-HRT users. Sener et al. (6) found the median age at diagnosis was 61.0 years for HRT users and 68.0 years for HRT nonusers (P <.001). They also found that HRT users more often had tumors that were <1 cm (P = .007), node negative (P = .033) and grade I (P = .016). Furthermore, compared to non-users, HRT users had a decreased risk of death (hazard ratio = .438, 95% confidence limit = .263 to.729, P = .002). It is not clear how many of these early detected tumors were the result of lead time bias.

Third, HRT is associated with an increased incidence of ER positive tumors (7–11). Without treatment, women with ER positive tumors have a better survival than those with ER negative tumors (12–14). Furthermore, endocrine therapy improves survival in ER positive tumors, including in those women who received HRT (15–18).

In summary, HRT use is associated with a younger age at diagnosis, an earlier stage at detection, and a high likelihood of responding to endocrine therapy – all of which contribute to better outcomes. It may be that HRT enhances our ability to image-detect early breast cancer rather than being a cause of breast cancer and it improves clinical outcomes. An implication of this view is that perimenopausal women may wish to start HRT, not only for its menopausal benefits, but also because of its benefit in the early detection of breast cancer.
